# Effectiveness of telesimulation on cardiorespiratory arrest for nursing students*

**DOI:** 10.17533/udea.iee.v41n2e07

**Published:** 2023-08-23

**Authors:** Francisco Mayron Morais Soares, Gleiciane Kélen Lima, Kirley Kethellen Batista Mesquita, José Erivelton de Souza Maciel Ferreira, Maria Jocelane Nascimento da Silva, Francisco Arnoldo Nunes de Miranda

**Affiliations:** 1 Nurse, Ph.D. Full Professorat University Center Inta - Campus Itapipoca, Itapipoca, Ceará, Brazil. Email: mayronenfo@gmail.com https://orcid.org/0000-0001-7316-2519 University Center Inta Itapipoca Ceará Brazil mayronenfo@gmail.com; 2 Nurse, Master. Nurse at the Doutor José Frota Institute, Fortaleza, Ceará, Brazil. Email: gleicianeklima@gmail.com. https://orcid.org/0000-0001-9334-1936 Nurse at the Doutor José Frota Institute Fortaleza Ceará Brazil gleicianeklima@gmail.com; 3 Master nurse. Federal University of Ceará Fortaleza, Ceará, Brazil. Email: kirleybatista@gmail.com https://orcid.org/0000-0002-6459-36563 Universidade Federal do Ceará Federal University of Ceará Fortaleza Ceará Brazil kirleybatista@gmail.com; 4 Nurse, Master student. University of International Integration of Afro-Brazilian Lusophony, Redenção, Ceará, Brazil. Email: eriveltonsmf@live.com https://orcid.org/0000-0003-2668-7587 University of International Integration of Afro-Brazilian Lusophony Redenção Ceará Brazil eriveltonsmf@live.com; 5 Nurse, Master Student. University of International Integration of Afro-Brazilian Lusophony, Redenção, Ceará, Brazil. Email: jocelane.nascimento.silva@gmail.com https://orcid.org/0000-0003-1764-7460 University of International Integration of Afro-Brazilian Lusophony Redenção Ceará Brazil jocelane.nascimento.silva@gmail.com; 6 Nurse, Ph.D. Full Professor, Federal University of Rio Grande do Norte, Natal-RN, Brazil. Email: farnoldo@gmail.com https://orcid.org/0000-0002-8648-811X Universidade Federal do Rio Grande do Norte Federal University of Rio Grande do Norte Natal RN Brazil farnoldo@gmail.com

**Keywords:** educational technology, simulation training, students, nursing, tecnología educacional, entrenamiento simulado, estudiantes de enfermería, tecnologia educacional, treinamento por simulação, estudantes de enfermagem

## Abstract

**Objective:**

To evaluate the effectiveness of telesimulation on cardiorespiratory arrest to improve the performance of nursing students.

**Methods:**

This was an experimental study, whose sample consisted of 30 undergraduate nursing students from a Brazilian university. It was structured from two groups: an experimental (*n*=15) and a control (*n*=15). For both groups, expository classes and skills training were held. For the experimental group, a virtual clinical simulation scenario was implemented. Before the beginning of the interventions, a pre-test was applied and, after the end of this, a post-test was applied to evaluate the students' gain of knowledge and skills.

**Results:**

From the analysis of the total correct answers and the scores obtained in the pre-test and post-test, it was found that there was an improvement in the performance of both study groups. Regarding the averages of the points obtained, there was a statistically significant difference between the groups (*p*=0.001). The post-test score was significantly higher than the pre-test score in the intervention group (*p*=0.001).

**Conclusion:**

The virtual scenario developed proved to be superior in improving the performance of nursing students in managing cardiorespiratory arrest when compared to traditional teaching methods.

## Resumen

**Objetivo:**

Evaluar la eficacia de la telesimulación del paro cardiorrespiratorio para mejorar el rendimiento de los estudiantes de enfermería.

**Métodos:**

Estudio experimental cuya muestra consistió en 30 estudiantes de pregrado de enfermería de una universidad brasileña asignados a uno de dos grupos: uno experimental (*n*=15) y otro control (*n*=15). Para ambos grupos se realizaron clases expositivas y entrenamiento en habilidades. Para el grupo experimental se implementó un escenario de simulación clínica virtual. Antes del inicio de las intervenciones se aplicó un pre-test y, tras el final de la intervención, un post-test para evaluar la ganancia de conocimientos y habilidades por parte de los alumnos.

**Resultados:**

A partir del análisis del número total de respuestas correctas y de los puntajes obtenidos en el pre-test y en el post-test se encontró que hubo mejoría en el desempeño de ambos grupos de estudio. En cuanto al puntaje promedio obtenido hubo una diferencia estadísticamente significativa entre los grupos (*p*=0.001). La puntuación post-test fue significativamente mayor que la puntuación pre-test en el grupo de intervención (*p*=0.001).

**Conclusión:**

El escenario virtual desarrollado demostró ser superior en la mejoría del rendimiento de los estudiantes de enfermería que se enfrentan a una parada cardiorrespiratoria, en comparación con los métodos de enseñanza tradicionales.

## Resumo

**Objetivo:**

Avaliar a efetividade da telesimulação sobre parada cardiorrespiratória para a melhora do desempenho de estudantes de enfermagem.

**Métodos:**

Tratou-se de um estudo experimental, cuja amostra foi composta por 30 estudantes de graduação em enfermagem de uma universidade brasileira. Foi estruturado a partir de dois grupos: um experimental (*n*=15) e um controle (*n*=15). Para ambos os grupos, foram realizadas aulas expositivas e treino de habilidades. Para o grupo experimental foi implementado um cenário virtual de simulação clínica. Antes do início das intervenções, aplicou-se um pré-teste e, após o término desta, aplicou-se um pós-teste para avaliar o ganho de conhecimentos e habilidades por parte dos estudantes.

**Resultados:**

A partir da análise do total de acertos e das notas obtidas no pré-teste e no pós-teste, constatou-se que houve melhora no desempenho de ambos os grupos de estudo. Referente às médias dos pontos obtidos, houve diferença estatisticamente significante entre os grupos (*p*=0.001). A nota do pós-teste foi significativamente maior que a nota do pré-teste no grupo intervenção (*p*=0.001).

**Conclusão:**

O cenário virtual desenvolvido mostrou-se superior, na melhoria do desempenho de estudantes de enfermagem frente à parada cardiorrespiratória, em comparação aos métodos de ensino tradicionais.

## Introduction

The advancement of Communication and Information Technologies (ICTs) has brought numerous changes to the way of living, thinking and acting of today's society, especially in the area of education.^1^ With the incorporation of these technologies in the formatting of traditional classes, higher education has achieved great evolution. Mainly, during the confrontation of the COVID 19 pandemic, where institutions had to adapt to the new remote teaching model.^1^ In this space, the educational strategies associated with ICTs gained even more strength, such as telesimulation, which is considered a distance education strategy capable of allowing access to Clinical Simulation (CS), maintaining the characteristic of the controlled and safe environment. ^2^

There are several benefits associated with its implementation, especially the possibility of using it for the training of technical and practical skills.^2^ Its realization seeks to simulate the characteristics of a given clinical situation, aiming to reach the understanding of the real conditions of the clinical situation studied. The assembled environment seeks to recreate a reality, so that the student can practice, learn, test and evaluate.^3^ Given the dynamism of the knowledge acquisition process, the use of a single educational theory and a single teaching model is sometimes insufficient to advance learning.^4^ In order to design active courses and produce maturing clinical and reflective awareness of students, the educational spectrum and existing teaching models must be integrated.^4^

Simulation technologies, adapted to nursing education, were first introduced in the 1950s with low-fidelity models and gradually evolved into modern high-fidelity tools. Since then, these technologies have been widely adopted to support the acquisition of knowledge and technical development skills.^5^ Considering that the nurses are the professionals responsible, within the nursing team, for interpreting human responses and drawing up a care plan directed to the patients, nursing students must be trained and qualified, scientifically and intellectually, to properly manage real clinical situations.^4^ There are several clinical situations that require rapid and effective nursing and multiprofessional interventions, especially those classified as emergency, such as Cardiorespiratory Arrest (CRA). 

Cardiorespiratory arrest (CRA) is an emergency of vascular nature, responsible for a high rate of morbidity and mortality in the world. It can be understood as the interruption of circulatory and respiratory activity, leading the subject to present absence of palpable central pulse, irresponsibility and apnea.^6^ Thus, it is of paramount importance to provide fast and qualified care to the affected patient, because every minute in CRA, the patient has a 10% reduction in survival.^7^ Clinical simulation can be used, in nursing education, in order to raise the quality of training of new professionals, since it enables real experiences, from the interaction of the student with a controlled scenario that mirrors the reality of a clinical situation for the participant.^8^ In addition, it enables the participants to develop and improve their clinical reasoning, techniques and skills.^9^^,^^10^ In this context, the present study aimed to evaluate the effectiveness of telesimulation on cardiorespiratory arrest to improve the performance of nursing students

## Methods

This is a quantitative experimental study, consisting of two groups; an experimental group and a control group; formed by nursing students from a Brazilian educational institution. In the experimental study, subjects are randomly allocated to one or more treatment or comparison groups. The study seeks the relationship between phenomena, seeking to know if one is the cause of the other.^11^ Data collection was carried out in March 2021. The research took place at a private university in the state of Ceará, located in the Northeast of Brazil. The study population was composed of nursing students, from the third year, enrolled in the university chosen to carry out the study. The choice of educational institution was due to the convenience of the researchers. The sample size was estimated using a formula based on McNemar's Chi-square test. After applying the test, the values of 86.9% of study power and 30 participants were obtained. Initially, all students in the fifth semester were invited to participate in the study. After acceptance, they were sent to a reserved room for further clarification about the research and its stages. Subsequently, the inclusion and exclusion criteria were applied, and written consent was given. 

The inclusion criteria were: being regularly enrolled in the third year of the nursing course of the university selected to carry out the study, being aged 18 years or older, and without previous practical experience in the management of CRA. The exclusion criteria were: not complying with all stages of the study and/or not fulfilling the research instruments in their entirety. All students in the fifth semester were invited to participate in the study, of which 34 met the eligibility criteria. However, four students were excluded, according to the exclusion criteria presented. Of the 30 students selected for the study, 15 participated in the experimental group and 15 in the control group. 

Data collection was performed through four stages. The first stage consisted of a prior appointment to meet with students, present the research and sign the ICF. The students received an identification number and were randomized, simply by computer program, into two groups, namely: Control Group (CG), which participated in the training with traditionally used teaching strategies - dialogued expository class and simulated skills training; Intervention Group (IG), which also participated in the dialogued expository class and simulated skills training, however followed by the simulated virtual scenario - telesimulation. Then, the second stage was carried out, which consisted of a face-to-face meeting to instrumentalize the study and distribute the previous bibliography. The third stage dealt with the training of the data collection team, carried out before the teaching intervention. The data collection team consisted of the researcher, a nurse and the laboratory technician.

The fourth stage dealt with the performance of the experiment. Initially, an instrument was applied to identify the prior knowledge of all students about CRA (pre-test). Then, the experiment itself was carried out, detailed below. For both groups, the expository classes and skills training were built from the contents available in the discipline of Clinical Nursing I with themes of CRA and CPR, following the theoretical framework of the American Heart Association and the Ministry of Health of Brazil. For skills training, practical classes were made available with teachers of the aforementioned discipline in laboratories equipped with medium fidelity simulators (torso with feedback device). For this moment, the students were divided into groups of six and the duration was approximately 60 minutes.

For the experimental group, in addition, a virtual scenario of medium-fidelity clinical simulation was implemented, with the SimSave *software*, *with* a subscription acquired by the authors. For this, all simulation design guidelines were followed regarding teaching-learning objectives, fidelity, problem solving, student support and debriefing. ^5^ The simulation implemented with the IG was carried out in the institution's nursing laboratory, with a controlled CRA scenario, in a hybrid manner (using low-fidelity mannequins and a simulated patient). In the scenario, information was passed on about the clinical case and the expected conduct. In other words, students should conduct the simulated CRA scenario according to their previous knowledge (briefing). After the procedure, the simulation itself was performed, which consisted of a patient on CRA requiring resuscitation. This moment lasted up to ten minutes. Afterwards, we discussed the strengths and weaknesses in the provision of nursing care (debriefing). After the intervention, the assessment of the development of cognitive skills (post-test) was carried out. We chose to do so to minimize the factors that could influence the test (for example, the acquisition of knowledge through specific studies for the resolution of the post-test).^12^

It is important to note that both groups performed the tests (pre and post), which consisted of the same questions and order with 25 items on Basic and Advanced Life Support. The items of the instruments were taken from the American Heart Association reference ^13^, which were evaluated by the participants as true (V) or false (F). The data were organized in a Microsoft Office Excel 2013 spreadsheet and analyzed using the Statistical Package for the Social Sciences (SPSS) software, version 23.0. Analysis by protocol, descriptive analysis, and inferential analysis were performed. Data normality was verified using the Shapiro-Wilk test. To evaluate the intergroup mean differences, the Mann-Whitney test was applied. The hypothesis of differences in pre-test and post-test scores was tested using the Wilcoxon marked rank test. For all analyses, the significance level of p<0.05 was adopted.

The study was approved by the Research Ethics Committee (CAAE: 16140619.5.0000.5576). All research participants signed the Informed Consent Form, after being verbally informed about the objectives and procedures of the study.

## Results

**Figure 1:**
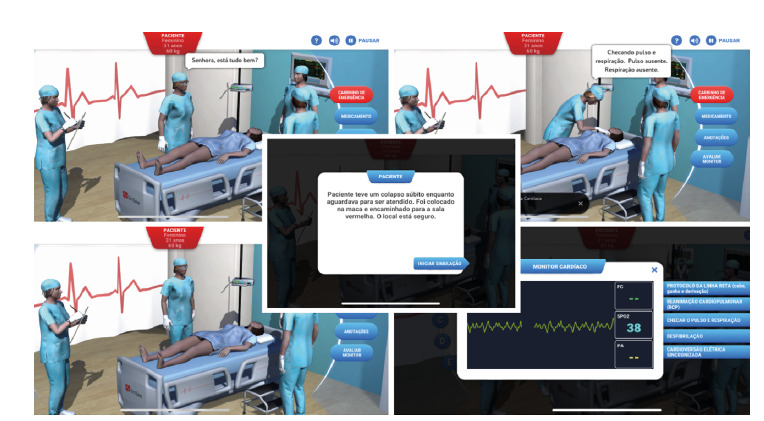
Virtual simulator screens in CRA scenario

Source: SimSave S *oftware*

Thirty nursing students of the seventh semester participated in the present study, of whom 20 (66.6%) were female, with a mean age of 20.6 years. Regarding the educational profile of these students, eight (26.6%) took an extracurricular course on the subject and five (16.6%) had taken a nursing technician course and had no previous professional experience in the role. Regarding the virtual scenario, SimSave *software* was used*,* with controlled environments on Basic Life Support (BLS) and Advanced Life Support (ALS). Thus, (Figure 1) presents the characteristics of student use.

Regarding the number of correct answers and errors in the pre-test and post-test, there was a minimum of 12 and a maximum of 14 correct answers in the pre-test; and in the post-test there was a minimum of 14 and a maximum of 24 correct answers. The topics with the highest error rates addressed the respiratory system and the compression *versus* ventilation ratio. And the topics with the highest rates of correct answers were the CRA, the automatic external defibrillator (AED) and the cardiovascular system. The correct answers and errors of both groups, as well as their scores, in the pre-test and post-test, were organized in the following table (Table 1). Considering that the groups were independent, the Mann-Whitney test was applied to verify whether the difference between the means of the groups was significant. There was an improvement in the performance of both groups based on the comparison of the total correct answers and the scores obtained in the pre-test and post-test, as shown in Table 1. In addition, the mean/median values and minimum scores of the intervention group were higher than those of the control group. Based on the results still presented in Table 1, there was a statistically significant difference between the intervention and control groups regarding the means of the posts obtained in the post-test (*p*=0.001).

**Table 1:** Intergroup performance for the CG and IG of the study

Variables	Groups		***p*-value**	ΔVar
	Intervention	Control		
Total pre-test hits	14.97	12.97	0.039	1.26
Total post-test hits	24.07	16.90	0.001	12.3
Pre-test note	6.22	5.78	0.039	
Post-test note	9.01	6.24	0.001	

Once a statistical difference in intergroup performance was identified in Table 2, the intragroup performance was analyzed, which is presented below. Based on the information described in Table 2, it is possible to state that there was a statistically significant difference between the intervention and control groups with regard to the means of the posts obtained only in the post-test (*p*=0.001). In both groups there was a positive evolution, which is when the post-test score is higher than the pre-test. In the intervention group, all participants had a positive evolution. In the comparison group, there were two negative outcomes and six ties when the correct answers and errors were compared in the pre-test and post-test, that is, three students obtained a post-test score lower than the pre-test and three obtained the same score in the pre and post-test. The Wilcoxon nonparametric test revealed that the post-test score was significantly higher than the pre-test score in the intervention group (*p*=0.001).

**Table 2:** Intragroup performance for the CG and IG of the study

Variables		Groups			
		Intervention		Control	
		*n*	Mean	*n*	Mean
Note 2 - Note 1*	Negative evolution^†^	0		2	3.50
	Positive evolution‡	15	8.49	7	6.04
	Tie§	0		6	
Statistics	Z^¶^	-2.919		-2.345	
	*p*-value	0.001		0.09	

*Note 2: Post-test; Note 1: Pre-test; †Note 2 < Note 1; ‡Note 2 > Note 1; §Note 2 = Note 1; ||Wilcoxon test; Result based on the averages of the posts with negative evolution.

## Discussion

Cardiorespiratory arrest CRA is one of the most important and prevalent emergencies faced by nursing professionals in their work environment, whether intra- or extra-hospital. The survival rate of individuals is closely related to the speed with which care is started, as well as to the qualification of professionals involved in care, so that the assistance provided is effective to reverse the patient's clinical condition.^14^ It is in this context that the development of clinical skills of nursing students is extremely relevant, since, most of the time, it is this professional, a nurse, who performs the first care, identifies that the patient is on CRA and already starts the cardiopulmonary resuscitation maneuvers.^2^ Nurses' clinical competence is crucial for providing accurate care to patients on CRA, and education plays a key role in improving this competence. In this sense, it is important to use interactive learning modalities, such as virtual clinical simulation, with valid scenarios, to encourage students to actively engage in patient care, in addition to providing significant benefits, such as improved knowledge, self-confidence, clinical performance, communication, critical thinking and clinical decision-making. Virtual clinical simulation provides a realistic and safe environment for students to practice and hone their skills.^14^

In this study, the successful implementation of an educational course based on telesimulation was described, which originated from the need to improve the teaching-learning of nursing students during clinical practice for patient care in CRA. This type of approach is an innovative method and distinct from the traditional teaching of nursing practices, arousing greater interest in students. It is a type of technology that favors student learning in different clinical situations, but in a controlled and safe scenario, which allows to err without causing negative repercussions to the patient.^12^ The results of this survey revealed a positive overview regarding content learning. Participants reported that this teaching experience added educational value above their learning when compared to traditional lectures, while it is more effective than standard learning exercises, being superior to conventional teaching methods. Corroborating these findings, a study found that most students agreed that the simulation contributed to the development of logical reasoning, the execution of teamwork, nursing techniques and procedures, in addition to assisting in autonomy and professional posture.^14^ This improvement after clinical simulation can be identified mainly among younger students, aged between 18 and 28 years, especially with regard to the acquisition of cognitive and practical knowledge, as well as self-confidence.^15^

Thus, the simulation is not only relevant to improve learning, it is able to raise the levels of satisfaction and self-confidence of nursing students who have or have not previous clinical experience, but it is important to note that the use of simulation does not exempt the student from clinical experience and contact with the real patient.^16^ In addition, it is noticed that as the semesters progress, students become more aware of the importance of experience in the clinical context, which may reflect on the ethics and responsibility of the student to seek to improve triple knowledge, skill and attitude.^14^ In view of this, clinical simulation is essential during the training process of nursing professionals assisting them in decision-making.^17^ The simulation is an experience that proves the need for it to be inserted in undergraduate courses so that there is early training of students, ensuring quality in teaching, as well as in patient care and professional training.^18^ In addition, it provides the opportunity to review errors that can be avoided in similar scenarios during professional practice, contributing to patient safety when in the real scenario.^19^

When considering that each student takes a different amount of time to assess the situation and make decisions about the conduct that will be necessary, the realistic scenario must be built based on the students' previous experiences, so the teacher needs to plan and organize the scenario based on a script that guides him in structuring the clinical simulation,^20^ especially when it comes to urgent and emergency situations. It is also noteworthy that the debriefing, used in this study, when carried out properly, after the simulations, has enabled nursing students to carry out associations with various knowledge, mainly related to the affective, cognitive and psychosocial dimensions, favoring the development of skills necessary for professional practice.^21^ In addition, through the debriefing, a study found that it was possible to work on nursing undergraduates to develop skills, as well as reflect on the simulated situation and the actions taken, improving skills, communication and professional attitude in the face of the emergency scenarios that were simulated.^22^

Thus, debriefing is important as it synthesizes, without judging the participants, the main topics that should occur during a given clinical simulation, favoring the assimilation of knowledge and the development of technical and interpersonal skills.^23^ Therefore, it is a phase of clinical simulation that comes to aggregate and consolidate teaching-learning about the simulated clinical scenario. Although this method has been shown to be beneficial for teaching, it was possible to observe in the present study, barriers and challenges during the implementation of the telesimulation course, mainly related to the minimum requirements that must be achieved or the resources that must be obtained to carry out a course of this magnitude, namely: telecommunications equipment, simulation resources and personnel experienced in conducting a simulation-based course, choice of the software to be used and connection to the internet.

Other challenges pointed out by the literature are related to the lack of financial resources for the structuring of scenarios and realistic makeup, information on the relevance of evidence-based practice, timely during the work to carry out the training, more commitment of educational institutions, as well as problems of technological infrastructure and lack of continuing education for the improvement of educators, in order to make teaching in undergraduate courses in health increasingly better.^22^^-^^24^ In view of this, to alleviate these challenges, greater engagement of universities is necessary to implement this method and to train teachers and those responsible for conducting the simulations, in addition to partnering with other institutions to try to reduce the production costs of the realistic scenario. Thus, the application of telesimulation in cardiorespiratory and cerebral arrest is an effective active methodology in the teaching-learning of students, providing a safe practice in the face of the clinical emergency scenario. In this sense, nursing students can test their clinical reasoning, in addition to improving their knowledge, skills and attitude that make them more self-confident to act in the professional environment, since they have already experienced a certain clinical situation through simulated practice.

In recent years, mainly due to the COVID-19 pandemic and the restrictions that face-to-face teaching has had, several innovative and alternative teaching methods have been developed and improved to assist in the teaching-learning of students, especially those in the health area, one of them being clinical simulation. Thus, this study contributes to the planning of clinical simulations by teachers for the teaching of nursing students, given that this strategy is able to immerse the students in the scenario of their future professional experience, enabling the improvement of their critical-reflective reasoning and their decision-making capacity. 

As a conclusion of the study, it is clear that the scenario of telesimulation focused on cardiorespiratory arrest was superior to the conventional teaching methods adopted, being, therefore, a plausible tool to be used by teachers in teaching in the health area, especially in nursing education. This technology proved be favorable in the acquisition of technical skills and abilities of nursing students, sharpening their critical reasoning, providing greater security for the students and the patients, in addition to making the students the protagonists of their learning.

As for the limitations of the study, obstacles stand out for the implementation of the telesimulation course due to the unavailability of equipment in the educational institution and people qualified to operationalize the system of the software used. For future studies, it is suggested the integration of nursing students with those from other areas such as medicine, to simulate the dynamics of interaction between these professionals in a clinical scenario of cardiorespiratory and cerebral arrest.
